# Supplementary material for: Energetic-Materials-Driven Synthesis of Graphene-Encapsulated Tin Oxide Nanoparticles for Sodium-Ion Batteries
Source: Materials (Basel). 2021 May 14;14(10):2550. doi: 10.3390/ma14102550 (PMC8157060; doi:10.3390/ma14102550)
Supplement: Supplementary file 1 [file materials-14-02550-s001.zip › materials-1211933-supplementary.pdf]

materials

Supplementary Materials

Energetic-Materials-Driven Synthesis of Graphene-Encapsulated Tin Oxide Nanoparticles for Sodium-Ion Batteries

Yingchun Wang [1], Jinxu Liu [1], Min Yang [1], Lijuan Hou [2], Tingting Xu [2], Shukui Li [1,3], Zhihua Zhuang [1] and Chuan He [1,*]

[1] School of Materials Science and Engineering, Beijing Institute of Technology, Beijing 100081, China; 18361232309@163.com (Y.W.); liujinxu@bit.edu.cn (J.L.); 15326716870@163.com (M.Y.); bitleesk@bit.edu.cn (S.L.); zhihua0802@163.com (Z.Z.)
[2] Key Laboratory of Materials Physics of Ministry of Education, Zhengzhou University, Zhengzhou 450052, China; houlijuan0815@163.com (L.H.); xutt@zzu.edu.cn (T.X.)
[3] Department of Materials Science and Engineering, Shenzhen MSU-BIT University, Shenzhen 518172, China
* Correspondence: chuan.he@bit.edu.cn; Tel.: 010-6891-3937

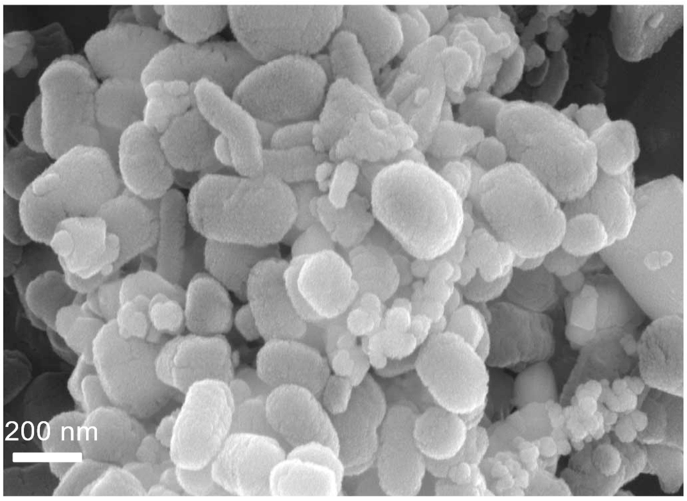

Figure S1. SEM image of the SnO_2 particles.

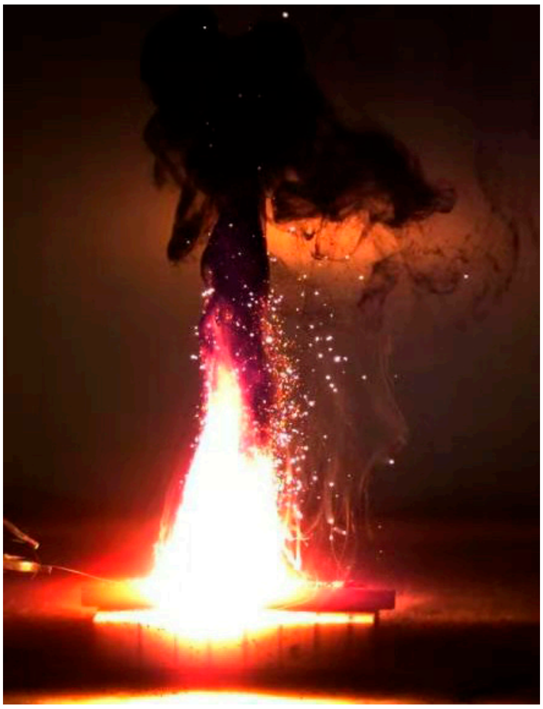

Figure S2. The photograph of the combustion process of the PTFE/Si/SnO_2 mixture in the ambient atmosphere recorded using a high-speed camera.

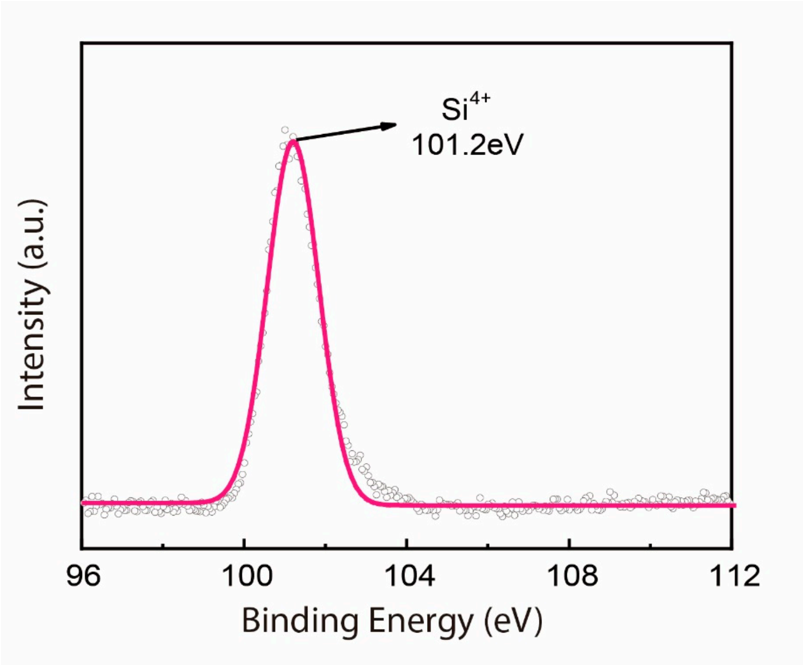

Figure S3. The high-resolution Si 2p spectrum of the Gr-SnO_2 nanoparticles.

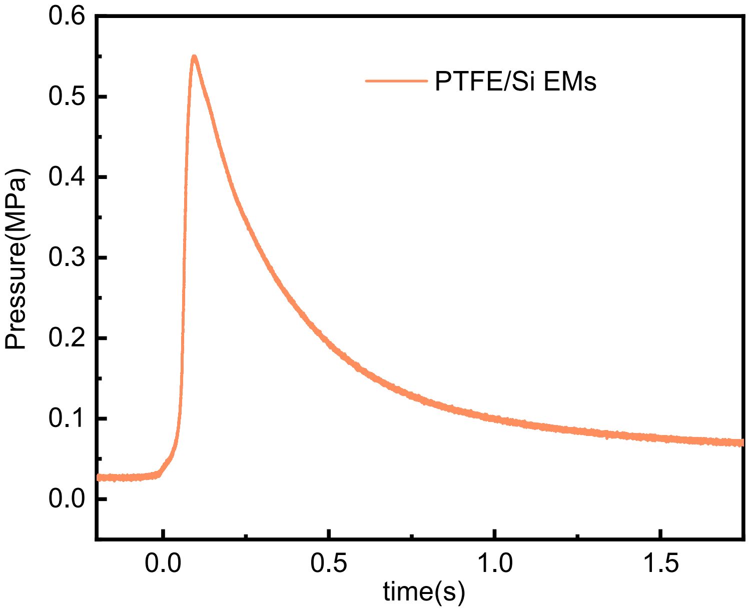

Figure S4. Curve of the pressure versus time of 0.5g of 70PTFE-30Si (wt.%) in closed bomb with 220 ml internal volume. The size of Si particles is 500 nm.
